# Analysis of healthcare service utilization after transport-related injuries by a mixture of hidden Markov models

**DOI:** 10.1371/journal.pone.0206274

**Published:** 2018-11-08

**Authors:** Nazanin Esmaili, Massimo Piccardi, Bernie Kruger, Federico Girosi

**Affiliations:** 1 Faculty of Engineering and IT, University of Technology Sydney, Ultimo, NSW, Australia; 2 Capital Markets Cooperative Research Centre, Sydney, NSW, Australia; 3 Transport Accident Commission, Geelong, VIC, Australia; 4 Translational Health Research Institute, Western Sydney University, Penrith, NSW, Australia; Texas A&M University College Station, UNITED STATES

## Abstract

**Background:**

Transport injuries commonly result in significant disease burden, leading to physical disability, mental health deterioration and reduced quality of life. Analyzing the patterns of healthcare service utilization after transport injuries can provide an insight into the health of the affected parties, allow improved health system resource planning, and provide a baseline against which any future system-level interventions can be evaluated. Therefore, this research aims to use time series of service utilization provided by a compensation agency to identify groups of claimants with similar utilization patterns, describe such patterns, and characterize the groups in terms of demographic, accident type and injury type.

**Methods:**

To achieve this aim, we have proposed an analytical framework that utilizes latent variables to describe the utilization patterns over time and group the claimants into clusters based on their service utilization time series. To perform the clustering without dismissing the temporal dimension of the time series, we have used a well-established statistical approach known as the *mixture of hidden Markov models* (MHMM). Ensuing the clustering, we have applied multinomial logistic regression to provide a description of the clusters against demographic, injury and accident covariates.

**Results:**

We have tested our model with data on psychology service utilization from one of the main compensation agencies for transport accidents in Australia, and found that three clear clusters of service utilization can be evinced from the data. These three clusters correspond to claimants who have tended to use the services 1) only briefly after the accident; 2) for an intermediate period of time and in moderate amounts; and 3) for a sustained period of time, and intensely. The size of these clusters is approximately 67%, 27% and 6% of the number of claimants, respectively. The multinomial logistic regression analysis has showed that claimants who were 30 to 60-year-old at the time of accident, were witnesses, and who suffered a soft tissue injury were more likely to be part of the intermediate cluster than the majority cluster. Conversely, claimants who suffered more severe injuries such as a brain head injury or anon-limb fracture injury and who started their service utilization later were more likely to be part of the sustained cluster.

**Conclusion:**

This research has showed that clustering of service utilization time series is an effective approach for identifying the main user groups and utilization patterns of a healthcare service. In addition, using logistic regression to describe the clusters in terms of demographic, injury and accident covariates has helped identify the salient attributes of the claimants in each cluster. This finding is very important for the compensation agency and potentially other authorities as it provides a baseline to improve need understanding, resource planning and service provision.

## Introduction and background

Transport injuries around the world often result in significant physical and psychological impairments and reduced quality of life [1, 2]. Every year, road traffic accidents cost most countries in excess of 3% of their gross domestic product (GDP), and between 20 and 50 million people suffer non-fatal injuries, with many incurring a consequent disability [3]. In the case of Australia, transport accidents are the second leading cause of hospitalized injuries and injury-related deaths [4], with massive health and financial impact [5]. In 2016 alone, nearly half a million motor vehicle accidents occurred in Australia, of which 1, 295 resulted in death (an increase of 7.5% compared to 2015), 32, 300 in serious injuries and 224, 104 in minor injuries requiring medical treatment [5]. Planning and providing adequate compensation to the sufferers of transport injuries is an ongoing challenge at national level. Insights into the burden of injury in specific injury groups may be provided by a quantitative analysis of the utilization of health services in the months and years following the accident [38]. A useful source of data to investigate health service utilization after accidents are the personal compensation datasets of insurers and compensation organizations [6, 7, 9]. These datasets make it possible to inspect the patterns of service utilization and their evolution over time from data of health service usage (e.g., number of visits to a specialists) at any desired level of aggregation (individual, group etc).

In the state of Victoria, Australia, the Transport Accident Commission (TAC) provides state-wide coverage of treatment, rehabilitation, vocational and disability benefits to individuals injured in land-based transport accidents [10, 38]. Despite the progress brought in by road safety programs, motor vehicle accidents still cause about 7, 800 serious hospitalizations per year in the state [10, 11]. The TAC possesses time series of all healthcare service compensation payments made to its claimants under the compensation scheme. Each time series records all the service utilizations made by a claimant and is identified by a unique claim ID. At its turn, each utilization includes the date of the service, its cost and its type, categorized according to three, increasing levels of detail. In addition to the service utilization data, the TAC also collects additional data about the claim, such as the age and gender of the claimant, the mode of transportation at the time of accident, what type of injuries were sustained, and so forth, grouped into the broader categories of demographic, injuries and accident data. In the terminology of data analytics, such extra data are commonly called “covariates” (or features, or attributes).

Grouping claimants based on their healthcare service utilization following a transport injury could prove beneficial in many ways. In the first place, it is likely to shed some light on the health “trajectory” of the claimamts. In the second instance, it could help improve resource planning and provide a model against which any future system-level interventions can be evaluated [38]. However, with the exception of severely-injured patients [8, 12–14], there seems to exist little published information regarding patterns of healthcare utilization following transport injuries [38]. In particular, to the best of our knowledge, there are no works in the literature exploring the temporal dimension of the service utilization.

In this research, we investigate patterns of healthcare utilization following transport injuries using de-identified compensation data provided by the TAC for the state of Victoria in Australia. Claimants were included in the study if they had lodged a claim in 2009 and their utilization data were collected until nine years after the accident. The measure of utilization was provided in the dataset as the number of monthly visits to service providers such as psychologists, psychiatrists, physiotherapists, chiropractors, practitioners, surgeons etc. Given that mental health is a priority for the compensation agency, in the rest of this paper we will illustrate our approach using time series of psychological services utilization. From these premises, the main goals of this study are to 1) cluster the claimants into homogeneous, distinct patterns of service utilization, and 2) characterize the clusters in terms of demographic, injuries and accident covariates to identify which types of claimants are likely to exhibit specific utilization patterns. To perform the clustering while properly taking into account the temporal order of the observations, we have employed a well-established sequential statistical approach known as the *mixture of hidden Markov models* (MHMM). Following the clustering, we have employed multinomial logistic regression to explain the cluster membership in terms of the covariates. Lastly, but not less importantly, this study should be regarded as a general methodology that can be immediately applied to any type of services and user pool.

## Data

The TAC provided two de-identified transport-related injury claim datasets spanning 2009 through to 2017 for claimants who lodged a claim in 2009. In Victoria, Australia, the compensation of psychology services for victims of transport accidents was established by a Government Act in 1986 [15] and retained to date by the Transport Accident Regulations of 2017 [16]. Therefore, there has been no affecting legislation changes during the observation period.

The first dataset contains one record for every compensation claim received by the TAC in 2009. This record consists of the information required for the management of the claim, including demographics (gender, current age, age at accident), accident-related data (accident date, claimant security risk type, claim development month, number of claims, road user type), and injury type. The injury type articulates over fatal, brain head, severe acquired brain injury (ABI), concussion, degloving, burns, spinal, amputation, quadriplegia, paraplegia, nerve damage, soft tissue, dislocation, internal injuries, sprain strains, limb fractures, non-limb fractures, contusion abrasion, sight, and other injuries. The second dataset includes longitudinal data of service utilizations and payments for 9, 328 unique claimants (1, 048, 576 total service utilizations), spanning January 2009-October 2017 (106 months). Each service utilization is labeled with three service categories at increasing level of detail (e.g., in order: hospital—rehabilitation; rehabilitation—private hospital; and rehab—physiotherapy). These categories have 30, 75 and 226 unique values each, respectively, across all the data. These two datasets were integrated for the analysis using the claim ID, and the information on payments was regarded as out of scope for the analysis. The Ethics Committee of University of Technology Sydney (UTS) and the Transport Accident Commission (TAC) approved restricted research use of these dataset (UTS Human Research Ethics Committee ETH182331).

The analysis we present in this paper can be carried out using any of the three service categories or their combinations. Since the third category is the most detailed, we have used it to select a specific service, and, given the importance of mental health for the TAC, we have chosen “psychology” to illustrate our model with. By “psychology service utilization” we mean an office visit to a psychologist. For this service, the longitudinal data contain 788 unique claimants for a total of 22, 523 service utilizations. In the following, all results refer to this service.

**Fig 1:**
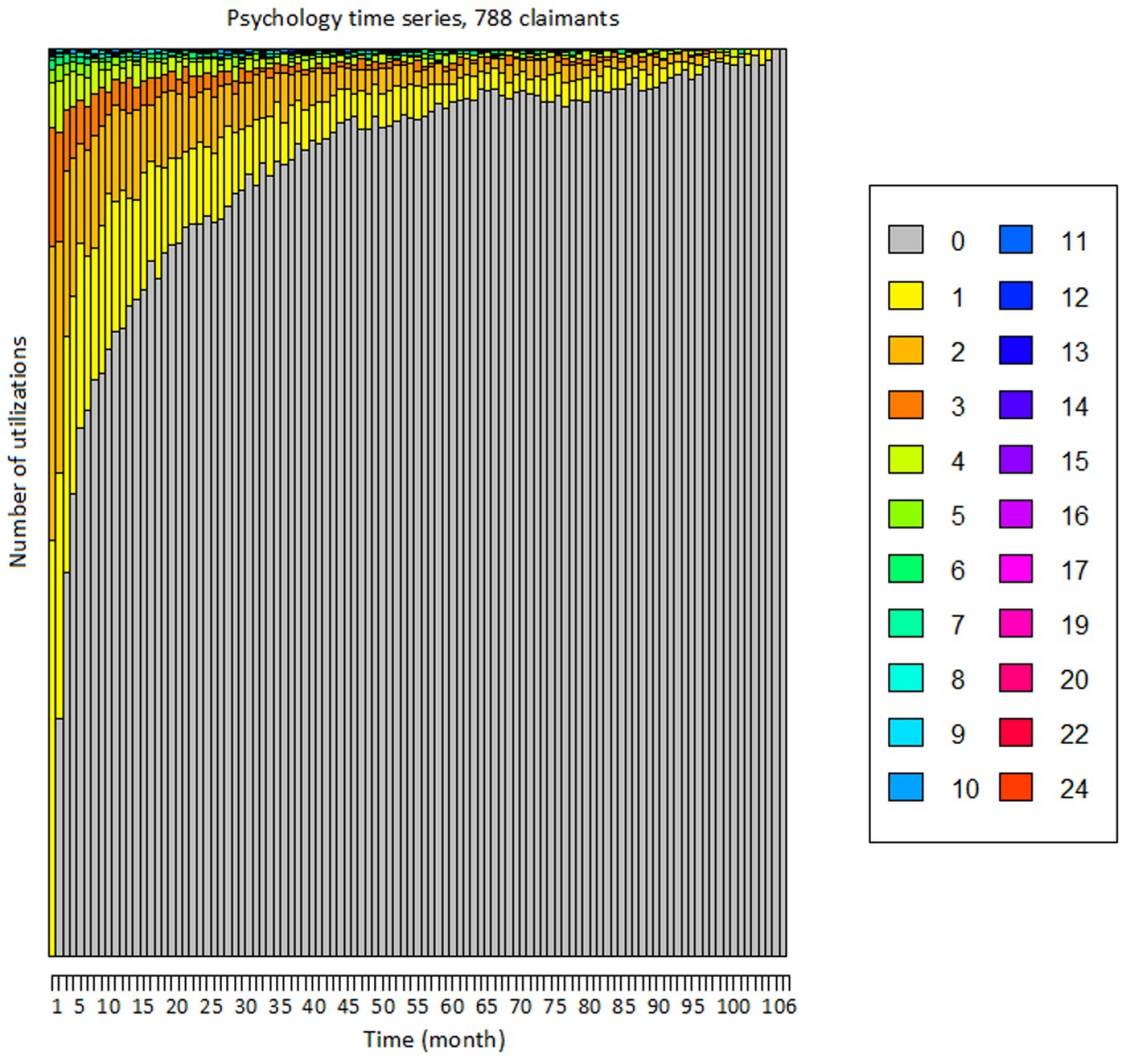
“Stacked plot” of the number of monthly utilizations of the psychology service. This figure shows the 788 times series as a “stacked plot. The height of each colored bar is proportional to the number of time series with that given number of utilizations.

We have built 788 time series with unique claim IDs by aggregating the number of utilizations by month. The first element of the time series is the month of first utilization, and the last element is the month of last utilization. Alternative alignments are possible; for instance, by the accident date. To take the accident date into account in our model, we have added the number of days between the accident date and the first utilization of the service as an additional covariate. The time series have variable length, from a minimum of 2 months to a maximum of 106, and on average approximately 29 months. The few (i.e., 6) time series lasting exactly 106 months are likely truncated by the finite length of the dataset. The number of service utilizations per month ranges over 0, 1, ⋯, 20, 22, and 24 and it has been displayed with a unique color through the entire paper (e.g., zero is grey, one is yellow, and so forth). Fig 1 offers a visualization of all the 788 times series as a “stacked plot”. In the figure, the height of each colored bar is proportional to the number of time series with that given number of utilizations. For instance, in the first month there is a large number of claimants with one utilization (yellow bar), fewer but still many with two (light orange bar), and so on. Conversely, toward the right end side of the plot almost all clients have zero (grey bar) utilizations. Table 1 shows the main statistics for the demographic, accident and injury covariates for the claimants who used the psychology service. In the analysis, we have only considered covariates that have at least 10% coverage of the sample.

**Table 1:** Main statistics for the demographic, accident and injury covariates for the psychology service.

Varaibles	Percentage	Yearly service utilization mean (SD)
Gender		
Female	52%	4.5 (8.4)
Male	48%	3.8 (6.6)
Age group (at accident)		
< 30-year-old	35%	4.7 (9.7)
30-40-year-old	22%	4.1 (6.9)
40-50-year-old	21%	4.2 (6.5)
50-60-year-old	13%	3.2 (3.7)
> 60-year-old	9%	3.6 (6.3)
Role in transport accident		
Driver	48%	3.8 (6.2)
Passenger	23%	4.1 (8.5)
(Motor/) Cyclist	16%	3.8 (4.8)
Pedestrian	12%	6.1 (12.9)
Witness	3%	4.2 (4.2)
Injuries		
Brain head (N)	76%	3.1 (4.6)
Brain head (Y)	24%	7.4 (12.6)
Concussion (N)	81%	3.7 (5.9)
Concussion (Y)	19%	6.1 (12.3)
Internal (N)	71%	3.4 (5.0)
Internal (Y)	29%	6.0 (11.6)
Soft tissue (N)	47%	4.4 (9.1)
Soft tissue (Y)	53%	3.9 (5.8)
Dislocation (N)	81%	3.8 (6.5)
Dislocation (Y)	19%	5.6 (10.9)
Sprain strains (N)	76%	3.8 (7.2)
Sprain strains (Y)	24%	5.1 (8.8)
Limb fractures (N)	63%	3.9 (6.4)
Limb fractures (Y)	37%	4.5 (9.3)
Non-limb fractures (N)	62%	3.2 (4.0)
Non-limb fractures (Y)	38%	5.6 (11.1)
Contusion abrasion (N)	33%	4.6 (7.5)
Contusion abrasion (Y)	67%	3.9 (7.7)

## Methodology

The modeling of sequential data (i.e., data that come in sequences such as our service utilization time series) requires significantly different assumptions from the more common case of independent samples. Given a generic time series, noted as (*x*_1_, *x*_2_, …, *x*_*t*_, …, *x*_*T*−1_, *x*_*T*_) or as *x*_1:*T*_ more compactly, there generally exists no analytical expression for its probability, *p*(*x*_1:*T*_). The common approach is to resort to *factorized* models and Markov assumptions to express the probability as the product of simpler terms, e.g. $p\left(x_{1:T}\right)=p\left(x_{1}\right)\prod_{t=2}^{T}p\left(x_{t}|x_{t-1}\right)$ as in an AR(1) autoregressive model [17]. These assumptions are often realistic and have permitted the implementation of accurate predictive models in many fields [17].

The *hidden Markov model* introduces a further assumption to improve the descriptive capability of a factorized model [18]. The assumption posits that each sample, rather than depending directly on the immediately previous samples, only depends on a categorical latent variable called the “state”. Such a variable completely encapsulates the state of the model at any given point in time. In turn, the model’s state evolves based on a set of transition probabilities. Therefore, the variables involved in a hidden Markov model (HMM) consist of the sequence of observations, *x*_1:*T*_, and the corresponding sequence of states, noted as *y*_1:*T*_. The factorized joint probability of the observations and the states is expressed as:

$$\begin{array}{c} p\left(x_{1:T},y_{1:T}\right)=p\left(y_{1}\right)\prod_{t=2}^{T}p\left(y_{t}|y_{t-1}\right)\prod_{t=1}^{T}p\left(x_{t}|y_{t}\right) \end{array}$$ (1)

The terms on the right hand side of Eq (1) fully define an HMM and include: 1) the probability of the initial state, *p*(*y*_1_); 2) the probability of transitioning from the state at time *t* − 1 to the state at time *t*, *p*(*y*_*t*_|*y*_*t*−1_); and 3) the probability of observing value *x*_*t*_ when in state *y*_*t*_, *p*(*x*_*t*_|*y*_*t*_). Such factors are commonly referred to as initial, transition and observation probabilities and form the *generative model* of the HMM. Each state variable is a latent categorical variable with an arbitrary number of values, let us say, *N*: therefore both *p*(*y*_1_) and *p*(*y*_*t*_|*y*_*t*−1_) can be modelled by conventional categorical distributions. Conversely, a single “observation” can consist of any combination of categorical and numerical values: therefore, term *p*(*x*_*t*_|*y*_*t*_) can be modelled using corresponding joint categorical/numerical distributions.

To give an intuition of an HMM at work, consider a toy example of a sequence of six observations, *x*_1:6_ = {5, 3, 7, 0, 3, 0}. We assume this HMM to have *N* = 2 distinct states that we qualitatively describe as “high utilization” (*H*) and “low utilization” (*L*). A plausible state sequence for these observations is *y*_1:6_ = {*H*, *H*, *H*, *L*, *L*, *L*}. This means that the first three samples have been generated by a state where higher values are more likely, while the remaining three from a state with lower likely values. Also notice that a same value (3 in this case) can be generated with non-null probability from multiple states. This implies that the states cannot be trivially inferred one by one from the range of the observations; rather, they have to be inferred at once from the entire observation sequence. Given Eq (1), this inference is formally expressed as:

$$\begin{array}{c} y_{1:T}^{*}=\underset{y_{1:T}}{\mathrm{argmax}}\;p\left(x_{1:T},y_{1:T}\right) \end{array}$$ (2)

An efficient, well-known solution for the state inference is provided by a dynamic programming algorithm known as the Viterbi algorithm [18]. Given a sequence of observations, the inferred sequence of states can be seen as a “macroscopic view” of a subject’s evolution over time. For this reason, such views have been added to Section Experiments and Results.

A number of other canonical problems exist for an HMM, including deriving various marginal probabilities from (1) and finding the optimal parameters for all its factor distributions under a maximum-likelihood framework. All these problems enjoy proven, computationally-efficient algorithms which have made the HMM a popular model for the modeling of data sequences. Among others, HMMs have been used in computer vision [19], signal processing [20], natural language processing [21], financial prediction [22], gene finding [23] and RNA editing [24].

An HMM can be estimated by maximizing its likehood over a given set of “training” observation sequences. The estimated model will reflect the main trend of its training data: for instance, if a large number of the sequences contain low values and infrequent changes, the model will shape the probability distributions around these cases (i.e., low observation values, rare state transitions). For this reason, the model itself can be seen as the dominant pattern in the training data. Therefore, in the case of a more diverse set of training sequences, an immediate extension could be to employ *multiple* HMMs to fit the multiple dominant patterns in the set.

In principle, one could first cluster the time series using an off-the-shelf clustering algorithm, and then fit an HMM to each cluster. However, this approach would suffer from the limitations of the original clustering algorithm, and the estimated HMMs might not well describe the “uncertain”, boundary cases. Another limitation of this approach is that it assigns each time series to one and only one cluster (hard membership). In alternative to this approach, it is possible to fit all the HMMs optimally at once over all the times series using a *mixture of hidden Markov models* (MHMM), a specialized instance of the mixture model in statistics [25]. An MHMM is fully defined by the following joint probability:

$$\begin{array}{c} p\left(x_{1:T},y_{1:T},z\right)=p\left(z\right)p\left(x_{1:T},y_{1:T}|z\right)=p\left(z\right)p\left(y_{1}|z\right)\prod_{t=2}^{T}p\left(y_{t}|y_{t-1},z\right)\prod_{t=1}^{T}p\left(x_{t}|y_{t},z\right) \end{array}$$ (3)

where *z* is a categorical variable that indexes the clusters. Eq (3) simply states that the model consists of a prior probability for the clusters, *p*(*z*), and multiple HMMs whose initial, transition and observation probabilities are specific to the cluster. By choosing the number of HMMs, let us say, *M*, one chooses the number of dominant patterns that the MHMM is able to describe. Even more interestingly, such a mixture model can be fit on the training data using the same maximum-likelihood framework of the single model. The resulting model automatically “groups” the training sequences according to their closest dominant pattern and, as such, an MHMM provides an ideal, principled tool for the clustering of time series [25].

An MHMM has two parameters requiring external tuning: the number of HMMs in the mixture, *M*, and the number of states in each HMM, *N* (this value is usually shared by all the HMMs in the mixture even if, in principle, it is possible to set it individually). A typical procedure for the setting of these parameters starts from their minimum value (i.e., 2) and increases them in unit steps until a satisfactory trade-off between the model’s likelihood and its complexity is reached. Common trade-offs include the Bayesian information criterion (BIC) [26], the Akaike information criterion (AIC) [27] and the use of eye judgment. In this paper, we have used both eye judgement and BIC to select the external parameters.

### Multinomial logistic regression of cluster membership

After clustering, we apply multidimensional logistic regression to explain the cluster membership based on the the demographic, injury and accident covariates described in Section Data. Using external covariates to explain the clustering results allows us to describe what typical profiles of claimants are associated with specific service utilization behaviors.

Multinomial logistic regression is the logistic regression framework for multinomial responses, that are categorical variables that can take more than two values (in our case, the number of the clusters, *M* = 3). In multinomial logistic regression, one of the responses is chosen to serve as reference and a separate logit model is built for each of the remaining *M* − 1 responses to compare them with the reference. Typically, the response accounting for the largest share of the sample is chosen as the reference. Multinomial logistic regression is a prime investigation analysis in healthcare applications (see [28–30] for a few examples).

### General use of the applied methodology

The methodology employed in this paper for the analysis of psychology services utilization can be applied, substantially unaltered, to any other service or service combination. An MHMM is in fact a highly flexible model that can be used to cluster time series of univariate or multivariate observations, as well as categorical or numerical, or mixed. It also offers many other advantages: 1) it uses a proper temporal model to describe the patterns and form the clusters; 2) it maximizes a proven optimality criterion (the likelihood function); 3) it is not restricted to assigning each time series to one cluster only; rather, it can assign it to multiple clusters in proportion to their probabilistic memberships (soft membership), and 4) it produces a latent state representation that can shed further light on the clusters. A potential weakness of this method is that the number of states in the HMMs needs to be either chosen manually or estimated with an external validation approach such as BIC or AIC [26, 27]. Even if the number of states is estimated optimally, there is a chance that the states may overfit the given observations, leading to poor fitting of new samples. However, in this application the data are all available at the beginning of the study, and the models could be easily refitted should new data be acquired.

At its turn, multinomial logistic regression can always be applied on the resulting clusters to gain an understanding of their membership. The set of attributes is unrestricted and the approach can be applied with any number of clusters. Using MHMM first and multinomial logistic regression after ensures that the clusters are formed purely based on the patterns of service utilization, and the attributes are only used to describe their memberships. It would be, of course, possible to use the service utilizations and the attributes jointly to produce an alternative clustering, but their respective effects would be mixed up and challenging to interpret.

## Experiments and results

### Analysis of the utilization of the psychology service

Our time series consist of observed service utilizations which are assumed to be probabilistic functions of “hidden” utilization levels which evolve over time. This assumption brings two main advantages to our study: 1) it properly takes into account the temporal order of the data, and 2) it models the individual claimants as transitioning through varying utilization levels (“hidden states”). Both features are very important to ensure an accurate description of the service utilization behaviors. In order to determine the number of the clusters and the number of the states in each cluster as required by the model, we have carried out initial experiments with 2, 3, 4 and 5 clusters in combination with 2, 3, and 4 states per cluster. Using a combination of eye judgment and BIC for the selection, we have determined that 3 clusters with 3 states each provided the best trade-off for these data. The three clusters contain, respectively, 528, 211 and 49 members (approximately 67%, 27% and 6% of the population). Based on the dominant utilization trend in each cluster, we have named them as “brief” (utilization), “intermediate”, and “sustained”, respectively. A similar descriptive naming can be attempted for the states in each cluster by examining some of their numerical attributes such as their average duration or their range of utilizations. By examining the typical numbers of utilizations associated with each of the nine states, we have decided to name them according to the following five labels: “zero”, “low”, “medium”, “high”, and “very high”. While states with the same label in different clusters are not formally equivalent, they are comparable.

**Fig 2:**
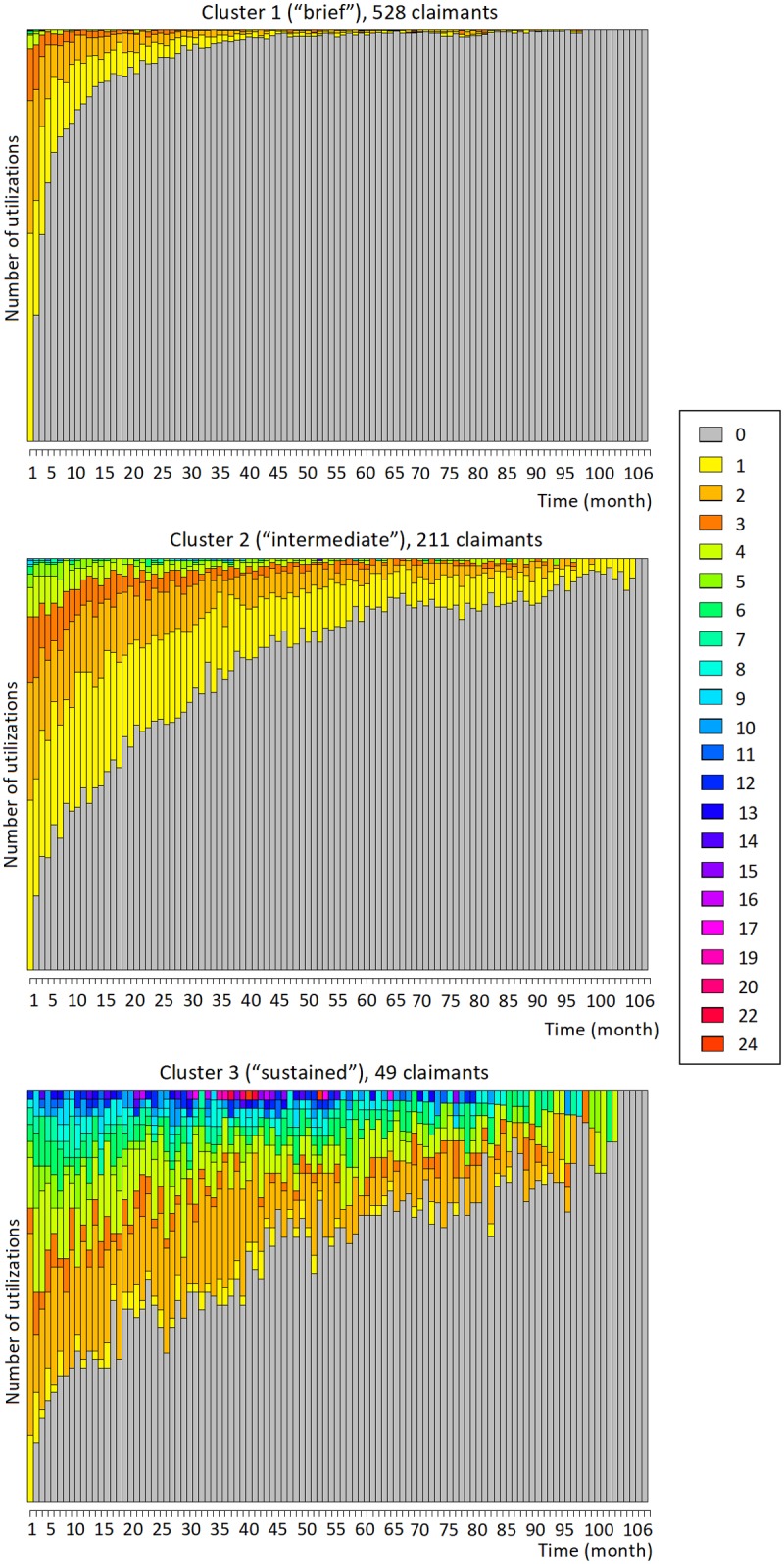
Stacked plots of the number of monthly utilizations of the psychology service for cluster 1 (528 claimants), cluster 2 (211 claimants) and cluster 3 (49 claimants). Based on the trends in the plots, we qualitatively describe these clusters as “brief”, “intermediate”, and “sustained”.

**Fig 3:**
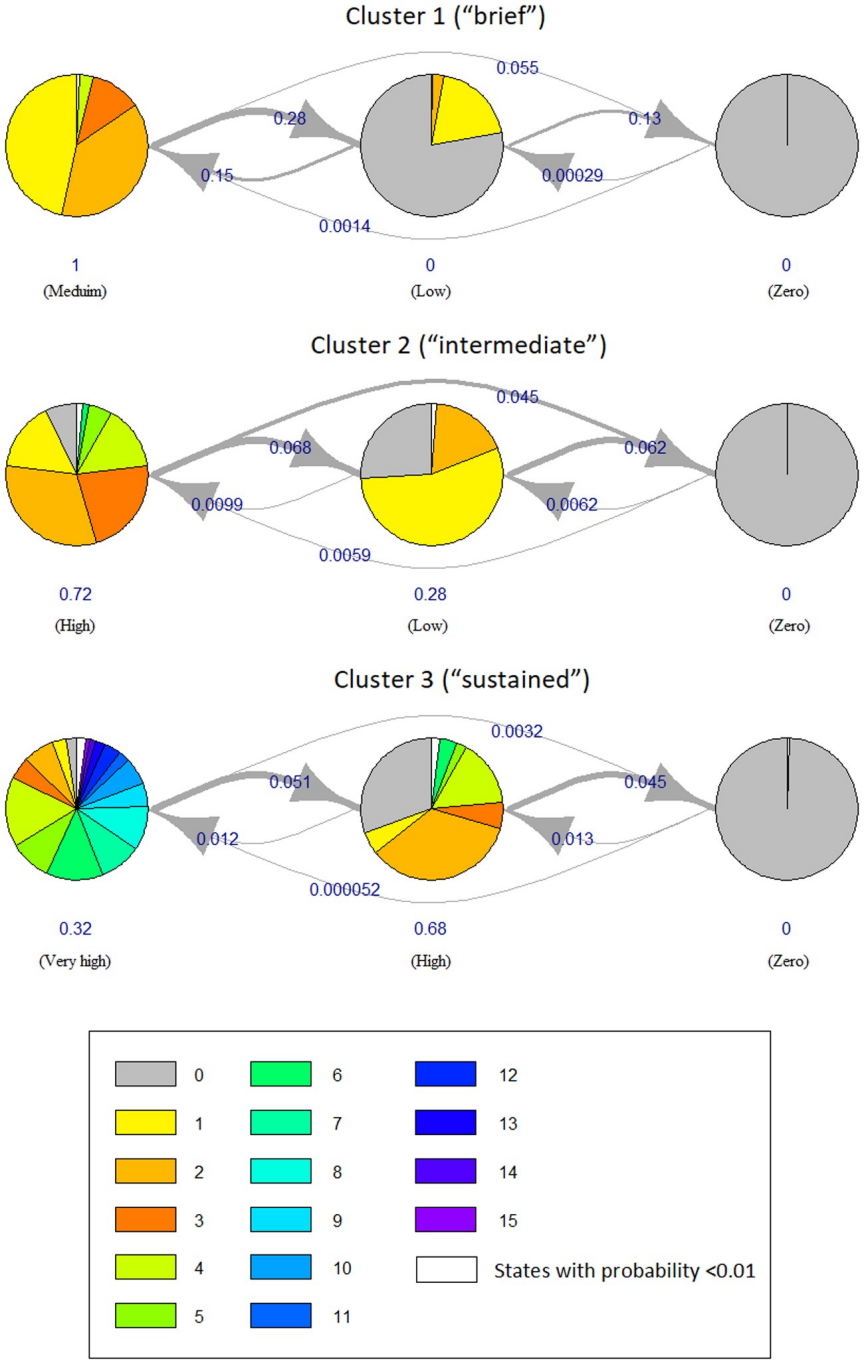
Stacked “state paths” for the three clusters of the psychology service. This figure shows the stacked “state paths” (i.e., the traversed sequences of states) of the claimants in each cluster. These plots confirm the different utilization trends in the three clusters.

**Fig 4:**
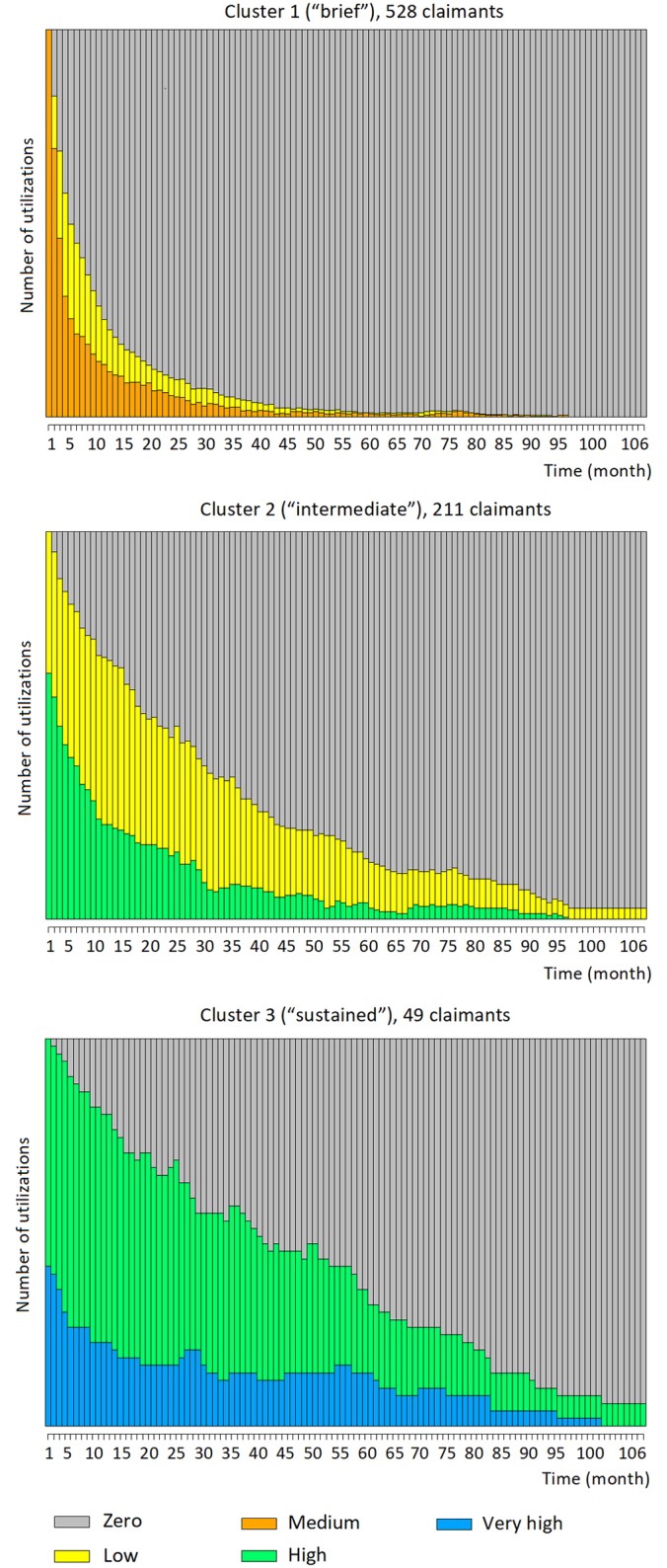
HMM state diagrams for the three clusters of the psychology service. Each state of each HMM is represented by a pie chart. The number of utilizations associated with the state are displayed as slices of the pie, with the size of each slice proportional to how frequent each number appears. The pies are connected by edges which represent how long the state typically lasts, and how frequently it instead changes to another state (i.e., the transition probabilities).

Several results for each cluster are plotted in the following Figs 2, 3 and 4. Fig 2 shows the stacked plots of service utilizations separately for each cluster, while Fig 3 shows the stacked “state paths” (the sequence of states traversed by a claimant) of the claimants in each cluster. These figures show unequivocally that the three clusters correspond to different typical amounts of service utilizations. Fig 2, top, shows that cluster 1 generally contains time series with low utilization. In the beginning, the utilization level is typically between 1 and 3 per month, then it quickly drops to reach 0. At months 4 and 20, respectively, more than 50% and 90% of the time series have already reached zero. The trend in cluster 2 is somehow similar, but the drop in service is less rapid: for instance, at months 4 and 20, respectively, only 20% and 50% of the time series have reached zero. The final cluster (cluster 3) contains claimants with higher utilization levels: at the beginning of the time series, a significant fraction of the claimants utilize the service even more than 10 times per month, and such amounts of utilizations are maintained for almost the entire observation period. Analogous considerations are suggested by the state plots displayed in Fig 3: the claimants in cluster 1 tend to start with a brief “medium” level of utilization, but quickly drop to “low” and “zero”. Conversely, more than half of the claimants in cluster 2 start with a “high” utilization level to then decrease to “low” and eventually “zero”, but more slowly compared to cluster 1. Lastly, 40% and 60% of the claimants of cluster 3 start with “high” and “very high” utilization levels, respectively. They then gradually transition toward “high” and “zero” utilizations; yet, at month 50, nearly 50% of the claimants are still at a “high” or “very high” utilization level.

Another informative visualization of an HMM is offered by its “state diagram”. In this visualization (Fig 4), each state of an HMM is represented by a pie. The numbers of utilizations associated with the state are displayed as slices of the pie, with the size of each slice proportional to how frequent each number appears. The pies are connected by edges which represent how long the state typically lasts, and how frequently it instead changes to another state (i.e., the transition probabilities). The higher the transition probability, the thicker the stroke of the edge and the more frequent is the transitioning from the state to the other. Fig 4 shows the state diagrams for the three clusters of the psychology service. To make the plot clearer, the numbers of utilizations with low frequencies (less than 0.01) have been combined into a single slice (white). Consistently with Figs 2 and 3, also Fig 4 shows that:

- cluster 1 (i.e., “brief”) transitions between “medium”, “low” and “zero” levels of utilization. The transitions toward decreasing levels are more frequent since the corresponding *transition probabilities* (i.e., the edges in the plot) are high (0.28 from “medium” to “low” and 0.13 from “low” to “zero”, respectively); however, transitions back from “low” to “medium” are also significant (probability of 0.15);
- cluster 2 (“intermediate”) typically transitions from the “high” level to “low” and “zero”, but less frequently (the corresponding transition probabilities are only 0.068 and 0.062, respectively);
- cluster 3 (“sustained”) typically stays at level “very high” for a while to then transition to “high” (probability of only 0.057). It then stays at “high” for a long time to eventually transition to “zero” (probability of only 0.045).

### Comparison with other clustering approaches

To probe our analysis further, we have compared our approach—clustering by MHMM—with other partition-based clustering approaches (algorithms) for time series, in particular the widely-adopted *partitioning around medoids* (PAM), *clustering large applications* (CLARA) and *fuzzy C-means* (FCM). Each of these algorithms divides a dataset into *M* groups (clusters) of observations, where the value for *M* is chosen beforehand. For a fair comparison, we have applied PAM, CLARA and FCM to exactly the same data and with the same number of clusters. We briefly describe these three algorithms in the next paragraph; however, the reader is referred to more detailed explanations in [31, 32] and [33].

PAM searches for *M* representative medoids among the time series in the dataset. A medoid is defined as the time series whose average distance to all the other time series in a cluster is minimal. PAM’s goal is to find *M* medoids such that the sum of the distances of the time series in the dataset to their closest medoid is minimized. The approach iterates over two steps: build and swap. In the build step, the medoids are determined from the current clusters, while in the swap step the time series are assigned to their closest medoid. This process is guaranteed to converge to a stable configuration of clusters and medoids. CLARA follows a similar approach to PAM, but finds the medoids over only a small sample of the time series (we have set the sample’s size to 50). It repeats the sampling and clustering processes a pre-specified number of times in order to minimize the sampling bias, and eventually selects the clustering results of minimal distance. At its turn, FCM is a soft clustering algorithm that, unlike PAM and CLARA (yet, similarly to MHMM), is not restricted to assigning each time series to one and one cluster only. Rather, it can assign it to multiple clusters by varying degrees of “fuzzy” (or soft) membership between 0 and 1 [31]. The time series closer to the centers of the clusters have higher degrees of membership than those near the borders, and influence more the determination of the centers.

The very notion of “good clustering” is relative and, ultimately, subjective. However, various quantitative indexes have found widespread use to measure the quality of clustering [33]. For this reason, in the rest of this section we use the silhouette and Dunn indexes to compare the four clustering algorithms (MHMM, PAM, CLARA, and FCM), showing that MHMM outperforms the other approaches over this task. Table 2 summarizes the results.

**Table 2:** Comparing the clusters obtained with MHMM, PAM, CLARA and FCM.

	MHMM	PAM	Clara	FCM
Size				
Cluster 1 (“brief”)	528	267	267	142
Cluster 2 (“intermediate”)	211	254	244	346
Cluster 3 (“sustained”)	49	267	277	300
Silhouette index				
Min.	-0.568	-0.502	-0.497	-0.418
1st Quartile	0.014	0.01	0.01	0.065
Median	0.326	0.147	0.145	0.244
Mean	0.216	0.131	0.132	0.212
3rd Quartile	0.466	0.301	0.307	0.379
Max.	0.507	0.422	0.437	0.504
Dunn index	0.019	0.012	0.014	0.014

With this technique, clusters 1, 2 and 3 have resulted, respectively, in 254, 267 and 267 members (roughly, 32%, 34% and 34% of the entire population). The size results in Table 2 shows that MHMM and FCM have been able to identify three uneven clusters (a “normal”, a “less frequent” and a “rare” cases) whereas PAM and CLARA have partitioned the time series over clusters all of approximately the same size, which seems a priori undesirable. In terms of mean silhouette index, MHMM and FCM have been significantly better than the other two methods, with MHMM (0.216) slightly above FCM (0.212). Eventually, MHMM has reported the highest Dunn index (0.019). Overall, MHMM has proved the most performing of the compared clustering methods.

**Fig 5:**
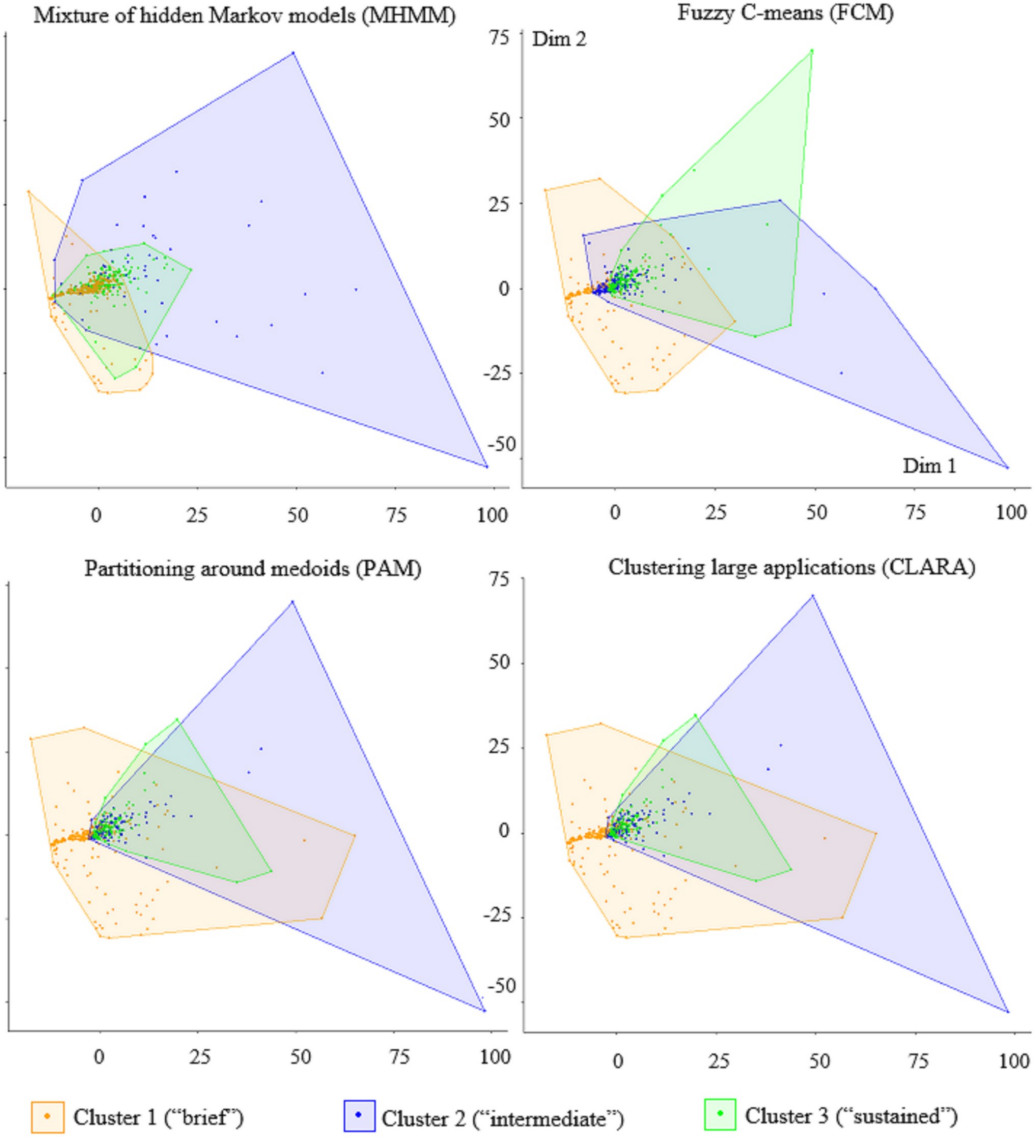
Comparing the clusters obtained with MHMM, PAM, CLARA and FCM MHMM. The clusters are plotted in 2D using multidimensional scaling.

In addition, Fig 5 compares the clustering results of these two four methods using a popular projection technique, multidimensional scaling (MDS). MDS is a visualization technique that is able to approximate a whole time series as a point in 2D, and visualizing the clusters as regions. As shown in Fig 5, with PAM and CLARA both cluster 2 and cluster 3 heavily overlap with cluster 1, and cluster 2 is almost a complete subset of cluster 3. This is not desirable since we expect to be able to divide the claimants more neatly. FCM, too, shows a significant overlap between clusters 2 and 3 with cluster 1, as well as cluster 3 with cluster 2. Except for 14 data points, all the members of clusters 2 and 3 overlap with at least another cluster. On the contrary, with the MHMM the three clusters are far less overlapping and more sharply defined. This shows that the MHMM approach is a more suitable clustering technique for temporal data.

Many other methods are available for clustering time series. Among them, optimal matching, marked point processes, and autoregressive (AR)-based clustering [34–37]). However, the MHMM has a principled advantage over all of them as it allows modeling each cluster in terms of latent states and their transitions. This feature of the model has allowed us to identify the main levels of service utilization and infer them for each time series as they change over time. While this feature may contribute to the accuracy of the clustering, it can also prove a useful descriptor of the utilization behaviors, both at the cluster and individual levels.

### Multinomial logistic regression results

Table 3 shows the results from the multinomial logistic regression analysis using the three clusters as responses and seven variables as independent inputs (i.e., covariates). Cluster 1 is the largest (approximately 67% of the claimants) and therefore been used as reference cluster. The input variables are categorized into four types as *demographic* (gender, age group at accident), *injury* (brain head, soft tissue and non-limb fractures), *time* (“elapsed time”) and *accident* (role in transport accident). Non-limb fractures refer to fractures that are not in the arms or legs such as skull, spine and ribs fractures. The elapsed time has been defined as the time in years between the date of the accident and the date of first utilization of the service. As for the encoding of the inputs, the elapsed time is a numerical variable, all injuries and the gender are binary variables, and the role in transport accident and age group are categorical variables. For each binary and categorical input, one of their values is used as reference in the analysis and as such does not appear in Table 3. In the case of the binary variables, the reference value is the alternative; for the role in transport accident, the reference value is “pedestrian” and for the age group is “< 30-year-old”. Four models using different subsets of the covariates are reported in Table 3, with all the covariates being statistically significant for at least one of the clusters (the confidence level is indicated by the asterisks next to the regression coefficients). Instead, the remaining covariates (concussion, internal dislocation, sprain strains, limb fracture and contusion abrasion) have not proved statistically significant for any of the clusters and therefore they have not been reported in the table.

**Table 3:** Multinomial logistic regression for clusters 1, 2 and 3, using cluster 1 as the reference.

	Model (1)	Model (2)	Model (3)	Model (4)
2:(intercept)	−1.66*** (0.20)	−2.15*** (0.26)	−2.14*** (0.27)	−2.57*** (0.40)
3:(intercept)	−2.03*** (0.25)	−4.08*** (0.52)	−4.44*** (0.55)	−4.37*** (0.66)
2:Gender (male)	−0.31* (0.19)	−0.28 (0.19)	−0.28 (0.19)	−0.29 (0.20)
3:Gender (male)	0.13 (0.29)	−0.58* (0.34)	−0.64* (0.35)	−0.54 (0.36)
2:30-40 years	0.71*** (0.26)	0.69*** (0.26)	0.69*** (0.26)	0.65** (0.27)
3:30-40 years	−0.46 (0.37)	−0.27 (0.41)	−0.18 (0.42)	−0.10 (0.43)
2:40-50 years	0.84*** (0.26)	0.82*** (0.26)	0.81*** (0.26)	0.81*** (0.26)
3:40-50 years	−0.98** (0.46)	−0.50 (0.51)	−0.42 (0.52)	−0.39 (0.52)
2:50-60 years	0.63** (0.30)	0.65** (0.30)	0.65** (0.30)	0.60** (0.31)
3:50-60 years	−2.38** (1.03)	−2.06* (1.06)	−2.08* (1.07)	−2.04* (1.07)
2:>60 years	0.27 (0.37)	0.21 (0.37)	0.20 (0.37)	0.21 (0.38)
3:>60 years	−0.66 (0.55)	−0.72 (0.61)	−0.68 (0.62)	−0.75 (0.66)
2:Brain head		0.51** (0.24)	0.50** (0.24)	0.58** (0.24)
3:Brain head		2.88*** (0.45)	2.89*** (0.45)	2.90*** (0.46)
2:Soft tissue		0.59*** (0.20)	0.59*** (0.20)	0.63*** (0.21)
3:Soft tissue		−0.04 (0.36)	−0.08 (0.36)	0.02 (0.37)
2:Non-limb fractures		0.10 (0.21)	0.10 (0.21)	0.13 (0.21)
3:Non-limb fractures		1.23*** (0.41)	1.20*** (0.41)	1.28*** (0.42)
2:Elapsed time (years)			−0.01 (0.06)	−0.01 (0.06)
3:Elapsed time (years)			0.23** (0.09)	0.23** (0.09)
2:Driver				0.40 (0.35)
3:Driver				−0.55 (0.49)
2: Passenger				0.42 (0.38)
3: Passenger				0.01 (0.52)
2:(Motor/) Cyclist				0.43 (0.41)
3:(Motor/) Cyclist				−0.36 (0.58)
2: Witness				1.34** (0.54)
3: Witness				1.17 (1.23)

Note:

*p<0.1;

**p<0.05;

***p<0.01

Table 3 shows the following significant relations:

- **gender:** the differences in terms of gender were mild. In general, male claimants were less likely to be members of the “sustained” cluster than the reference “brief” cluster;
- **age:** in terms of age group, claimants who were 30 to 60-year-old at the time of the accident were more likely to be members of the “intermediate” cluster than the “brief” cluster. In addition, claimants who were 50 to 60-year old at the time of the accident were less likely to be members of the “sustained” cluster than the “brief” cluster. This trend was computed with respect to less-than-30-year-old as reference value;
- **injuries:** claimants who experienced a brain head injury or experienced non-limb fractures were significantly more likely to be members of the “sustained” cluster than the “brief” cluster; on the other hand, claimants who experienced a soft tissue injury were significantly more likely to be members of the “intermediate” cluster than the “brief” cluster;
- **accident:** in terms of role in the accident, witnesses were significantly more likely to belong to the “intermediate” cluster than the “brief” cluster. These trends were computed with respect to pedestrian as reference value. NB: in the TAC dataset, a witness is defined as anyone who was present at the accident scene other than the drivers, pedestrians and other parties involved in the accident.
- **time:** in terms of elapsed time, the later the claimants had their first utilization, the more likely they belonged to the “sustained” cluster compared to the “brief” cluster.

Overall, the results of the multinomial logistic analysis show that claimants who were 30 to 60-year-old at the time of accident, who were witnesses, and who suffered a soft tissue injury were more likely to belong to the “intermediate” cluster than the reference, majority cluster. Conversely, claimants who suffered more severe injuries such as a brain head injury or a non-limb fracture and who started their service utilization later were more likely to belong to the “sustained” cluster.

## Conclusions and future work

This research has aimed to identify distinct behaviors of service utilization, describe the characteristic differences between behavior groups and identify the dominant behavior of individual claimants with respect to the utilization of a healthcare service. To analyze the clients’ behaviors in terms of service utilization over time, we have used an authoritative statistical approach known as the mixture of hidden Markov models (MHMM). To conduct a case study, we have selected psychology as the service of interest and then optimally fitted an MHMM over the time series of 788 claimants. After fitting the model, each claimant has been assigned to its closest cluster. This step has led to the identification of three main, typical behaviors of utilization which we have referred to as “brief” (and low), “intermediate” (and lasting longer) and “sustained” (and high over a significant period of time). We have also provided extensive visualization of the results.

As the next step, we have investigated which profiles of claimants have tended to be associated with each cluster. To this aim, we have used multidimensional logistic regression to explain the cluster membership based on demographic, injury, time and accident covariates. From all the available covariates, we have identified some that appeared promising, including gender, the age at the time of accident, brain head, soft tissue, and non-limb fractures injuries, role in transport accident and the elapsed time from the date of the accident to the first utilization. The results have shown that several of these covariates are statistically significant for the cluster membership. The results indicate that compared to the “brief” cluster, the claimants who were 30 to 60-year-old at the time of the accident, had soft tissue injury as a result of the accident or had the role of witnesses in the accident were more likely to be members of the “intermediate” cluster than the “brief” cluster compared to claimants who were younger than 30, did not have soft tissue injury and had pedestrian as role in the accident, respectively. Again, compared to the “brief” cluster, claimants who had brain head and non-limb fractures injuries were more likely to be members of the “sustained” cluster. Conversely, those who were male and 50 to 60-year-old at the time of the accident were less likely to belong to this cluster. Moreover, the elapsed time was significant to assign claimants to the “sustained” cluster compared to the reference “brief” cluster.

The proposed approach is general and could be used by compensation agencies to predict ahead of time which claimants are likely to exhibit specific service utilization patterns. This ultimately provides the opportunity to design early, dedicated interventions aimed at improving the claimants’ treatment or improve offering of the services through packaging and provider agreements.

As future work, we are planning to investigate the correlation between different, yet related services. To do so, we envisage two lines of investigation: the first is to explore the relationship between multiple, related services; for example, the relationship between physical and psychological treatments. The second is to identify the services that are prevalent among specific pools of claimants; for example, the prevalence of psychiatric services among claimants with persistent pain. Overall, the main goal of this investigation is to better understand service utilization for improving recovery.
