# Correction: Analysis of healthcare service utilization after transport-related injuries by a mixture of hidden Markov models

**DOI:** 10.1371/journal.pone.0214973

**Published:** 2019-04-01

**Authors:** Nazanin Esmaili, Massimo Piccardi, Bernie Kruger, Federico Girosi

After publication of this article [1], concerns were raised that the references to the software packages used for this analysis had been omitted. The authors utilized Stata Statistical Software: Release 15. The reference is StataCorp. 2017. Stata Statistical Software: Release 15. College Station, TX: StataCorp LLC. The authors also utilized the seqHMM package package in R. The reference is: Helske S, Helske J. Mixture hidden markov models for sequence data: the seqhmm package in R. arXiv preprint arXiv:1704.00543. 2017 Apr 3.
